# Sleep quality in Chinese patients with rheumatoid arthritis: contributing factors and effects on health-related quality of life

**DOI:** 10.1186/s12955-016-0550-3

**Published:** 2016-11-16

**Authors:** Genkai Guo, Ting Fu, Rulan Yin, Lijuan Zhang, Qiuxiang Zhang, Yunfei Xia, Liren Li, Zhifeng Gu

**Affiliations:** 1 Department of Rheumatology, Affiliated Hospital of Nantong University, 20th Xisi Road, 226001 Nantong, People’s Republic of China; 2 School of Nursing, Nantong University, 19th Qixiu Road, 226001 Nantong, People’s Republic of China

**Keywords:** Rheumatoid Arthritis, Sleep quality, Quality of life, Depression, Disease activity

## Abstract

**Background:**

Poor sleep quality is common in rheumatoid arthritis (RA) patients and may lead to disease aggravation and decreased health-related quality of life (HRQoL). The increasing prevalence of poor sleep in RA patients is associated with adverse demographic, clinical, and psychological characteristics. However, there are currently no known reported studies related to the effects of sleep quality on HRQoL in RA patients from China. This cross-sectional study aims to evaluate the contributors of poor sleep and the effects of sleep quality on HRQoL in Chinese RA patients.

**Methods:**

A self-report survey was administered to 131 RA patients and 104 healthy individuals using the Pittsburgh Sleep Quality Index (PSQI) for sleep quality. RA patients completed the Hospital Anxiety and Depression Scale for anxiety and depression, the 28-joint Disease Activity Score for disease activity, the 10 cm Visual Analog Scale for pain, the Health Assessment Questionnaire-Disability Index for functional capacity and the Short Form 36 health survey for HRQoL. Blood samples were taken to gain some biochemical indicators (e.g., erythrocyte sedimentation rate, C-reactive protein, rheumatoid factor, and anti-cyclic citrullinated peptide). Independent samples t-tests, Chi square analysis, logistic regression modeling and linear regression were used to analyze these data.

**Results:**

Our results found that the prevalence of poor sleep (PSQI ≥ 5) was 78.6% and the mean global score of PSQI was 7.93 (SD 3.98) in patients, which were significantly higher than the controls (18.7% and 3.88 (SD 1.89), respectively). There were significant correlations among synthetic disease-modifying antirheumatic drugs, erythrocyte sedimentation rate, pain, disease activity, functional capacity, anxiety/depression and sleep quality in RA patients. Meanwhile, logistic regression models identified disease activity and depression as predictors of poor sleep quality. Poor RA sleepers had impaired HRQoL than good RA sleepers, and sleep quality was independently and significantly associated with social function and mental components summary.

**Conclusions:**

The majority of Chinese RA patients suffered from poor sleep, which significantly impairs their HRQoL. The data suggested the need for holistic assessment and management of RA patients and the importance of objective interventions to improve their sleep quality and finally to improve their HRQoL.

**Electronic supplementary material:**

The online version of this article (doi:10.1186/s12955-016-0550-3) contains supplementary material, which is available to authorized users.

## Background

Rheumatoid arthritis (RA) is a chronic, inflammatory, autoimmune disorder characterized by joint pain, joint swelling and destruction of synovial joints. RA influences about 0.5–1% of adults in industrialised countries and causes disability, pain, psychological problems and decreased quality of life [1–3]. Sleep quality is an important component of health-related quality of life (HRQoL), and it has been reported that sleep problems significantly negatively affect the HRQoL in patients with RA [4]. Therefore, identifying factors contributing to poor sleep quality and studying the effects of sleep quality on HRQoL in RA patients is of great importance.

Poor sleep is one of the most prevalent and burdening symptoms and can have debilitating effect on physical and/or mental function and health [5]. It has been reported that sleep problems occur in 54% − 70% of RA patients, including difficulty falling asleep, poor sleep quality, non restorative sleep, wakefulness, awakenings during the night and excessive daytime sleepiness [6, 7]. Importantly, poor sleep quality may contribute to greater pain, disease activity and mood disorders, creating a cascade of dysfunction for afflicted patients [8, 9]. It has been recognized that a number of influences, such as socioeconomic status, disease activity and psychological disorders may impact sleep health, which is common in the RA population [10, 11].

Despite the importance of sleep quality in RA patients, only a limited number of studies related to RA patients’ sleep quality have been conducted in China. The aim of this study is (1) to determine contributors of sleep quality in RA patients recruited from China; (2) to evaluate the effects of sleep quality on various dimensions of HRQoL in Chinese RA patients.

## Methods

### Participants

Patients who fulfilled the American College of Rheumatology (ACR) (2012) criteria for RA were recruited from the Affiliated Hospital of Nantong University from December 2014 and to December 2015 [12]. RA patients were outpatients or inpatients and healthy subjects were selected from a population attending for an annual examination. Patients were excluded based on either of the following: (1) they were less than 18 years old; (2) they did not complete questionnaires; (3) they had comorbidities (e.g., serious infections, or cardiac, respiratory, gastrointestinal, endocrine disease) that could influence disease activity; (4) they did not complete the measurements of disease activity and pain. Control subjects were excluded if they exhibited current or history of other systemic diseases or psychiatric disorders. The study was approved by the Ethics Committee of the Affiliated Hospital of Nantong University (2014-387), and written informed consents were obtained from all of the participants, according to the Declaration of Helsinki.

### Demographics and clinical characteristics

Demographic variables contain the following: body mass index (BMI), gender, age, marital status, education, occupation, and monthly income, smoking and alcohol use, which were obtained by a self-designed questionnaire. Clinical variables of disease duration, family history and medications use were obtained by asking patients or viewing medical records. Additional clinical variables of erythrocyte sedimentation rate (ESR), C-reactive protein (CRP), rheumatoid factor (RF), and anti-cyclic citrullinated peptide (anti-CCP) were examined at the time of investigation. ESR and CRP were assessed by the Westergren method (mm/h) and the nephelometric method (mg/L), respectively. RF was measured by immunoturbidimetry using Cobas integra RFII (Roche Diagnostics GmbH, Mannheim, Germany) and anti-CCP was measured by enzyme linked immunosorbent assay (ELISA) using DIASTAT™ (Axis-Shield Diagnostics, Dundee, UK) [13].

**Disease activity** was estimated with the valid and reliable 28-joint Disease Activity Score (DAS28), incorporating 28 swollen and tender joint counts, patient’s assessment of disease activity (0–100 mm VAS, where 0 = not active at all and 100 = extremely active), ESR (mm/h) and CRP (mg/L) [14].

**Pain** was measured by the Visual Analogue Scale (VAS) to characterize clinical pain severity. Patients were asked to rate their experience of pain during the last week, each on a VAS of 0–10, with a higher score indicating more severe pain [15].

**Functional capacity** was measured by using the Health Assessment Questionnaire -Disability Index (HAQ-DI). The HAQ-DI assesses the patient’s perceived difficulty in completing tasks in eight categories-dressing, arising, eating, walking, hygiene, reach, grip, and usual activities. The eight category scores were averaged into an overall HAQ-DI score and may range from 0 (no disability) to 3 (completely disabled). The Chinese HAQ-DI is a reliable and valid instrument for studies measuring disability of patients with RA [16]. “The test-retest reliability coefficient was 0.84. Between dimensions measured in HAQ-DI, the highest test-retest reliability was observed for walking (Spearman correlation coefficient rs = 0.80) and the lowest was for eating (rs = 0.54). The internal consistency of the scale using Cronbach's alpha was high at 0.86” [16].

### Assessment of psychological parameters

Psychological status was measured using the Hospital Anxiety and Depression Scale (HADS), which is divided into HADS-A and HADS-D, both of which containing seven intermingled items [17]. Each item had a 4-point Likert scale and was scored between 0 and 3. Scores for each subscale were constructed by summation, ranging 0 − 21. The Chinese version of HADS had acceptable internal consistency and test-retest reliability, with a Cronbach alpha of 0.85 and intraclass correlation coefficient of 0.90, respectively [18].

### Assessment of self-reported life quality

Participants’ health status was assessed using the Short Form 36 health survey (SF-36) in the past 4 weeks. It assessed eight domains (scores range from 0 to 100, with higher scores indicating better health status): physical function (PF); role limitations due to physical problems (RP); body pain (BP); general health perception (GH); energy/vitality (VT); social function (SF); role limitations due to emotional problems (RE); mental health (MH). Z-transformed and normalized domain scores were grouped into Physical Component Summary (PCS) and Mental Component Summary (MCS) [19]. The questionnaire was culturally adapted and translated into Chinese. “Convergent validity and discriminant validity were satisfactory for all except the social functioning scale. Cronbach’s α coefficients ranged from 0.72 to 0.88 except 0.39 for the social functioning scale and 0.66 for the vitality scale. Two weeks test-retest reliability coefficients ranged from 0.66 to 0.94” [20].

### Assessment of self-reported sleep quality

Sleep quality was examined by the Chinese version of Pittsburgh Sleep Quality Index (PSQI) during the month preceding, which includes 19 questions completed by the subject [21]. Previous studies have found that PSQI is a well-established, commonly used measure of sleep quality that has been applied across a range of adult populations, including those with RA and other medical conditions [22]. These 19 items were broken down into the following seven components: subjective sleep quality, sleep latency, sleep duration, habitual sleep efficiency, sleep disorders, use of hypnotics, and daytime dysfunction. The seven components were each scored from 0 (no difficulty) to 3 (severe difficulty), and summed, to give an overall score ranging from 0 to 21. Participants were dichotomized into a poor-sleep group if the PSQI was ≥5.0 and a good-sleep group if the PSQI was < 5.0 [23].

### Data collection

Resultant Chinese paper-based questionnaires were used to administer all instruments under physician supervision. Administration was performed in a single sitting, lasting from 15 to 20 min. Pain and DAS28 were evaluated by the same clinician for all patients. Results were added to a computer database by two research assistants and double checked against the original data prior to analysis.

### Data analysis

Descriptive statistics are provided using mean (±standard deviation (SD)) or number (percentage) depending on parametric distribution of measured variables. Potential demographic, clinic and psychological variables were screened using univariate tests of the group difference (poor sleepers versus good sleepers according to the PSQI), at a lenient level of significance without correction for multiple testing (alpha = 0.05). The differences in terms of continuous and categorical variables that are studied in RA patients who are grouped as poor sleepers and good sleepers were evaluated with the two-tailed *t* test and the chi-square test respectively, as well as the differences between the RA patients and the controls. All variables with a significant association with sleep quality by univariate tests were entered into a multiple stepwise logistic regression model with the dichotomous sleep quality measured by the PSQI as the dependent variable. The relations between quality of life and the other evaluation parameters were examined with Spearman rank correlation analysis. Linear regression analysis was used for each of the SF-36 components to determine independent association of sleep quality with quality of life in patients with RA after controlling for related factors, which have a significant association with each component of SF-36 by Spearman rank correlation analysis. Statistical significance was considered when *p* <0.05 (two-sided). All analyses were performed using SPSS version 20.0.

## Results

### Patient characteristics

As four RA patients and five healthy individuals did not complete the questionnaires, 131 RA patients (19 males and 112 females) and 104 healthy individuals (16 males and 88 females) were enrolled in the current study. Table 1 presents the baseline participant characteristics included in our study. Of the 131 RA patients included in this analysis, 85.5% were female and the mean (SD) age was 54.7(11.5) years. Almost 98.5% RA patients were treated with synthetic Disease-Modifying Antirheumatic Drugs (DMARD) and the mean (SD) ESR was 28.47 (27.64) mm/h. There was no significant difference in the age, gender, BMI, marital status, education, occupation, income/month, alcohol and smoke between the RA individuals and the controls (*p* > 0.05).

**Table 1:** Baseline characteristics of RA patients and the healthy controls

Variables	Cases (*n* = 131)	Controls (*n* = 104)	*p*
Gender, female, N (%)	112 (85.5)	88 (84.6)	0.79
BMI (kg/m^2^), Mean ± SD	22.5 ± 3.12	22.84 ± 2.81	0.432
Age (years), Mean ± SD	54.5 ± 11.5	54.77 ± 11.20	0.851
Monthly income (yuan), N (%)			0.973
<1000	67 (51.1)	55 (52.9)	
1000-3000	47 (35.9)	35 (33.7)	
3000-5000	13 (9.9)	10 (9.6)	
>5000	4 (3.1)	4 (3.8)	
Marital status, N (%)			0.411
Single	7 (5.3)	11 (10.6)	
Married	118 (90.1)	90 (86.5)	
Divorced	1 (0.8)	1 (1.0)	
Widowed	5 (3.8)	2 (1.9)	
Education, N (%)			0.571
≤9 years	93 (71.0)	70 (76.9)	
>9 years	38 (29.0)	34 (23.1)	
Occupation, N (%)			0.757
Employed	102 (77.9)	80 (80.8)	
Unemployed	29 (22.1)	24 (19.2)	
Alcohol use, yes, N (%)	24 (18.3)	19 (18.3)	1.000
Smoking use, yes, N (%)	10 (7.6)	10 (9.6)	0.642
Disease duration (years), Mean ± SD	8.7 ± 9.1		
Family history, yes, N (%)	10 (7.6)		
Medications use, N (%)			
NSAIDs	692 (52.7)		
Synthetic DMARDs	129 (98.5)		
Biologic DMARDs	11 (8.4)		
Glucocorticoid	62 (47.3)		
ESR (mm/h), Mean ± SD	28.47 ± 27. 64		
CRP (mg/L), Mean ± SD	16.61 ± 24.51		
RF positive, N (%)	102 (77.9)		
anti-CCP positive, N (%)	91 (69.5)		
DAS28, Mean ± SD	3.96 ± 1.44		
HAQ-DI, Mean ± SD	0.50 ± 0.61		
Total pain (VAS), Mean ± SD	4.32 ± 2.76		
Nocturnal pain (VAS), Mean ± SD	3.95 ± 3.22		
HADS-A, Mean ± SD	9.12 ± 2.79		
HADS-D, Mean ± SD	8.74 ± 2.46		

Data are means ± SD for continuous variables, or percentages for categorical variables

*p* values were obtained with the chi-square test for categorical variables and the two-tailed *t* test for continuous variables

*BMI* Body Mass Index, *NSAID* Nonsteroidal anti-Inflammatory Drugs, *DMARD* Disease-Modifying Antirheumatic Drugs, *ESR* Erythrocyte Sedimentation Rate, *CRP* C-reactive Protein, *RF* Rheumatoid Factor, *anti-CCP* anti-cyclic citrullinated peptide, *DAS28* Disease Activity Score in 28 Joints, *HAQ-DI* Health Assessment Questionnaire-Disability Index, *VAS* Visual Analog Scale, *HADS* Hospital Anxiety and Depression Scale, *SF-36* the Medical Outcomes Study Short Form 36, *PCS* Physical Components Summary, *MCS* Mental Components Summary, *PSQI* Pittsburgh Sleep Quality Index

### Self-reported sleep quality

In the present study, we found that 103 of the 131 patients with RA had a high risk for poor sleep quality. The results demonstrated that the prevalence of poor sleep (PSQI ≥ 5) in RA patients (78.6%) was higher than in the controls (18.7%) and the total PSQI score of patients with RA was significantly higher than that of the controls (7.93 ± 3.98 versus 3.88 ± 1.89; *p* < 0.01). Table 2 summarized the global score and the component scale scores of the PSQI for our RA sample in comparison with controls. The data showed that five of the seven components of PSQI in RA patients were significantly different from the controls: subjective sleep quality, sleep latency, habitual sleep efficiency, sleep disorders and use of hypnotics.

**Table 2:** Comparison of the components of PSQI in cases and controls

PSQI components score	Cases (*n* = 131)	Controls (*n* = 104)	*p*
Subjective sleep quality	1.23 ± 0.74	0.63 ± 0.73	<0.001
Sleep latency	1.70 ± 1.04	0.70 ± 0.74	<0.001
Sleep duration	0.80 ± 1.05	0.70 ± 1.00	0.451
Habitual sleep efficiency	1.16 ± 1.19	0.15 ± 0.41	<0.001
Sleep disorders	1.65 ± 0.69	0.90 ± 0.72	<0.001
Use of sleep medications	0.52 ± 1.04	0.06 ± 0.27	<0.001
Daytime dysfunction	0.88 ± 0.74	0.73 ± 0.80	0.148
Total	7.93 ± 3.98	3.88 ± 1.89	<0.001

Data are means (standard deviation) for continuous variables

*p* values were obtained with the two-tailed *t* test

### Differences between good and poor sleepers in RA patients

As shown in Table 3, a number of demographic, clinical and psychological variables were tested for possible differences between good sleepers and poor sleepers in patients. Poor sleepers had higher disease activity scores, higher level of ESR, severer total/nocturnal pain, higher degree of depression and anxiety, with a trend toward lower functional capacity as well, compared with good sleepers (*p* < 0.05). In addition, the percentage of synthetic DMARDs use in good sleep patients was significant higher than that of poor sleep patients (*p* < 0.05).

**Table 3:** Comparison between poor and good sleepers in RA patients

Variables	PSQI < 5 (*n* = 28)	PSQI ≥ 5 (*n* = 103)	*p*
Female	22 (78.6)	90 (87.4)	0.240
BMI (kg/m^2^)	22.88 ± 3.40	22.39 ± 3.01	0.347
Age (years)	54.79 ± 12.08	54.38 ± 11.45	0.872
Disease duration (years)	8.21 ± 8.99	8.83 ± 9.15	0.749
Monthly income (yuan)			0.141
<1000	12 (426.9)	55 (53.4)	
1000-3000	9 (32.1)	38 (36.9)	
3000-5000	6 (21.4)	7 (6.8)	
>5000	1 (3.6)	3 (2.9)	
Marital status			0.273
Single	1 (3.6)	5 (4.9)	
Married	25 (89.3)	94 (91.3)	
Divorced	1 (3.6)	0 (0)	
Widowed	1 (3.6)	4 (3.9)	
Education			0.240
≤9 years	17 (60.7)	76 (73.8)	
>9 years	11 (39.3)	27 (26.2)	
Occupation			0.798
Employed	21 (75.0)	81 (78.6)	
Unemployed	7 (25.0)	22 (21.4)	
Smoking use, yes	4 (14.3)	6 (5.8)	0.220
Alcohol use, yes	4 (14.3)	20 (19.4)	0.783
Family history	2 (7.1)	8 (7.8)	1.000
Medications use			
NSAIDs	17 (60.7)	52 (50.5)	0.396
Synthetic DMARDs	26 (92.9)	103 (100.0)	0.044
Biologic DMARDs	2 (7.1)	9 (8.7)	1.000
Glucocorticoid	11 (39.3)	512 (49.5)	0.396
ESR (mm/h)	19.14 ± 15.96	31.00 ± 29.39	0.006
CRP (mg/dl)	12.36 ± 25.00	17.7668 ± 24.37	0.336
RF positive	19 (67.9)	84 (80.6)	0.198
anti-CCP positive	19 (67.9)	72 (69.9)	0.821
DAS28	3.15 ± 1.39	4.17 ± 1.38	0.001
HAQ-DI	0.20 ± 0.38	0.52 ± 0.65	0.002
Total pain (VAS)	2.96 ± 1.86	4.69 ± 2.85	0.001
Nocturnal pain (VAS)	2.66 ± 2.54	4.30 ± 3.30	0.007
HADS-A	8.00 ± 2.46	9.42 ± 2.81	0.016
HADS-D	7.25 ± 1.76	9.03 ± 2.49	0.001

Data are means ± SD for continuous variables, or percentages for categorical variables

*p* values were obtained with the chi-square test for categorical variables and the two-tailed *t* test for continuous variables

*BMI* Body Mass Index, *NSAID* Nonsteroidal anti-Inflammatory Drugs, *DMARD* Disease-Modifying Antirheumatic Drugs, *ESR* Erythrocyte Sedimentation Rate, *CRP* C-reactive Protein, *anti-CCP* anti-cyclic citrullinated peptide, *RF* Rheumatoid Factor, *DAS28* Disease Activity Score in 28 Joints, *HAQ-DI* Health Assessment Questionnaire-Disability Index, *VAS* Visual Analog Scale, *HADS* Hospital Anxiety and Depression Scale, *SF-36* the Medical Outcomes Study Short Form 36, *PCS* Physical Components Summary, *MCS* Mental Components Summary, *PSQI* Pittsburgh Sleep Quality Index

### Determinants of self-reported sleep quality in RA patients

We used stepwise multiple logistic regression analysis to investigate predictors of sleep quality, as indicated in Table 4. We found that HADS-D(β = 0.340, *p* < 0.01) and DAS28 (β = 0.520, *p* < 0.01) were the predictors of sleep quality.

**Table 4:** Stepwise multiple logistic regression analysis of demographic, medical and psychological variables in relation to PSQI in RA patients

Predictors	β	SE	*p*	Exp(B)	95%CI	
					Lower	Upper
HADS-D	0.340	0.122	0.007	1.406	1.106	1.786
DAS-28	0.520	0.193	0.004	1.681	1.152	2.453

*p* values were obtained with stepwise multiple logistic regression analysis

*DAS28* Disease Activity Score in 28 Joints, *HADS* Hospital Anxiety and Depression Scale, *PSQI* Pittsburgh Sleep Quality Index

### Effects of self-reported sleep quality on HRQoL

Comparison of poor sleepers and good sleepers in terms of HRQoL was summarized in Table 5. Patients with poor sleep quality had significantly lower HRQoL in all domains of the SF-36 scale. The relations between HRQoL and the other evaluation parameters were examined with Spearman rank correlation analysis (Additional file 1: Table S1). Considering observed associations of demographic, disease-related and psychological factors with sleep quality, we conducted linear regression analysis for each of the SF-36 components to determine independent association of sleep quality with quality of life after controlling for related factors, which have a significant association with each component of SF-36 by Spearman rank correlation analysis. The results showed that sleep quality was independently and significantly associated with SF (β = -0.204, *p* = 0.012) and MCS (β = -0.158, *p* = 0.037). Sleep quality was not significantly associated with the remained components of SF-36 (Table 6).

**Table 5:** Comparison between poor and good sleepers in RA patients

Variables	PSQI < 5 (*n* = 28)	PSQI ≥ 5(*n* = 103)	*p*
PF	69.82 ± 28.07	54.27 ± 28.43	0.015
RP	41.07 ± 48.70	17.23 ± 32.09	0.020
BP	60.79 ± 24.72	44.91 ± 25.54	0.004
GH	54.86 ± 21.70	38.29 ± 19.72	<0.001
VT	63.39 ± 17.69	48.59 ± 18.03	<0.001
SF	75.89 ± 26.12	58.62 ± 26.55	0.003
RE	54.76 ± 46.45	31.39 ± 41.96	0.012
MH	71.29 ± 18.48	58.56 ± 17.56	0.002
SF-36 PCS	56.63 ± 24.63	38.68 ± 19.95	0.001
SF-36 MCS	66.33 ± 22.72	49.30 ± 20.27	0.001

*p* values were obtained with the two-tailed *t* test for continuous variables

*PF* physical function, *RP* role limitations due to physical problems, *BP* body pain, *GH* general health perception, *VT* energy/vitality. *SF* social function, *RE* role limitations due to emotional problems, *MH* mental health, *PCS* Physical Components Summary, *MCS* Mental Components Summary, *PSQI* Pittsburgh Sleep Quality Index

**Table 6:** Linear regression analysis of sleep quality on each of the SF-36 components

SF-36 components	β	*p*
PF^a^	0.057	0.471
RP^b^	-0.018	0.836
BP^c^	-0.060	0.383
GH^d^	-0.016	0.067
VT^e^	-0.097	0.255
SF^f^	-0.204	0.012
RE^g^	-0.151	0.091
MH^h^	-0.068	0.397
PCS^i^	-0.045	0.527
MCS^g^	-0.158	0.037

*p* values were obtained with multiple linear regression analysis

^a^controlled for income, disease duration, ESR, CRP, DAS28, total pain, nocturnal pain, HAQ-DI, and HADS. ^b^controlled for income, synthetic DMARDs, CRP, DAS28, total pain, nocturnal pain, HAQ-DI, and HADS. ^c^controlled for income, synthetic DMARDs, CRP, RF, DAS28, total pain, nocturnal pain, HAQ-DI, and HADS. ^d^controlled for age, alcohol, disease duration, synthetic DMARDs, DAS28, total pain, nocturnal pain, HAQ-DI, and HADS. ^e^controlled for income, disease duration, ESR, CRP, DAS28, total pain, nocturnal pain, HAQ-DI, and HADS. ^f^controlled for age, income, synthetic DMARDs, ESR, CRP, RF positive, anti-CCP positive, DAS28, total pain, nocturnal pain, HAQ-DI, and HADS. ^g^controlled for age, income, synthetic DMARDs, ESR, CRP, RF positive, DAS28, total pain, nocturnal pain, HAQ-DI, and HADS. ^h^controlled for occupation, income, ESR, CRP, DAS28, total pain, nocturnal pain, HAQ-DI, and HADS. ^i^controlled for income, disease duration, synthetic DMARDs, ESR, CRP, DAS28, total pain, nocturnal pain, HAQ-DI, and HADS

## Discussion

Sleep problems are common in patients with chronic diseases and the prevalence of poor sleep is high in RA patients compared to controls [24–26]. In the current study, 78.6% of RA patients suffered from poor sleep, and disease activity and depression were independent predictors of poor sleep quality in RA patients. What is more, poor RA sleepers had impaired HRQoL than good RA sleepers and sleep quality was independently and significantly associated with HRQoL. To our knowledge, this is the first examination of the effects of sleep quality on HRQoL in Chinese RA patients.

It has been reported that several factors of demographic, clinical and psychogenic variables have an influence on sleep quality [27–29]. In our study, patients with a PSQI score ≥5 were found to have significantly more frequently use synthetic DMARDs. However, Nicassio PM et al. examining sleep disturbance in RA patients have found no association between synthetic DMARDs and sleep quality [30]. While, one study has shown that YORA (age at disease onset ≤ 65 years) patients reported more sleep disorders and received significantly more synthetic DMARDs than LORA (age at disease onset > 65 years) RA patients [31]. In our present study, the majority of RA patients were under 65 years and 98.5% patients had synthetic DMARDs, which may account for the differences in our findings compared to other reported findings [30].

An increasing number of studies have indicated that psychological disorders are risk factors for sleep quality [4, 32, 33]. It has been reported that 20% − 30% of RA patients have significant mood problems [9, 34]. Psychological disorders especially depression aggravate the numerous health-related comorbidities that are related to RA such as disability and limitations in quality of life [35, 36]. In the present study, we also found that poor-sleep patients had significantly higher levels of HADS-A and HADS-D scores compared with good-sleep patients. Interestingly, logistic regression analysis indicated that depression was a major contributor to sleep quality, which is consistent with previous studies [28, 30]. The data suggested the need for systemic psychiatric screening and management and the importance of targeted interventions to help RA patients get rid of sleep problems [30]. At present, some non-pharmacological treatments, such as intermittent aerobic exercise, can relieve depression and anxiety, as well as improving sleep quality in RA patients [37]. Meanwhile, it is reported that sleep management including sleep education and cognitive-behavioral interventions can improve physical/mental wellness and sleep quality [38].

Previous study has found that disease activity has a vital effect on sleep quality in RA patients [33, 34]. Westhovens et al. examining sleep problems in patients with RA have found a positive and independent association between disease activity and sleep quality [29]. Our study has shown poor-sleep patients had significantly higher levels of disease activity compared with good-sleep patients. Importantly, logistic regression analysis indicated that disease activity is a major contributor to sleep quality, which is consistent with previous studies from Turkey and Korea [4, 27]. Additionally, the ESR was found to be associated with sleep quality in our study, which was in line with prior research reports [4]. It is possible that ESR more closely correlated with polysomnography parameters recorded during sleep hypoxic episodes and moderate and severe obstructive sleep apnea were independently associated with an elevated ESR [39]. Also, there was a positive correlation between sleep quality and pain in RA patients, which was also found in our study [32]. Unexpectedly, pain did not contribute to poor sleep quality in the logistic regression analysis of our study, which was different from a previous study [30]. The possible explanation may be that the active inflammatory disease and the resulting pain are treated and the arthritic process is stopped, which resulted in less influence than other variables on sleep quality [40]. Similar to the results of Wolfe et al., the data in the present study support that functional capacity is associated with sleep quality [32]. The inability to perform the daily activities may cause depression and lead to poor sleep quality in RA patients [41]. After administration of the IL-6 receptor antagonist tocilizumab in patients with RA, changes in PSQI score over time were correlated significantly with HAQ-DI changes and marginally with changes in fatigue [42]. Therefore, IL-6 may play an important role in the mechanism of sleep quality.

HRQoL is considered as one of the main outcome measures for RA patients and sleep quality represents a central component of HRQoL in RA patients [4]. Several studies have shown that individuals with RA have poorer quality of life compared to the general population, in both the emotional and physical domains [43, 44]. We found diminished HRQoL in poor sleepers in all dimensions of SF-36 compared with good sleepers. Association between sleep quality remained significant for SF and MCS after controlling for demographic, disease-related and psychological factors. It is of interest that RA patients had poorer HRQoL, including general health, physical health, mental health and sleep [45]. Associations of sleep problems with pain and fatigue in RA patients are also reported by some previous studies [46]. It must be noted that fatigue and pain in RA patients are multidimensional with physical and mental health. The HRQoL instrument we used in our study was not comprehensive in this regard, which may explain our results.

Though we reported the relationship between the demographic, clinical, psychological characteristics and sleep quality and the effects of sleep quality on HRQoL in Chinese patients with RA, we also had several limitations: (1) We just measure sleep quality and psychological factors with self-report questionnaires. (2) Since the sample size was rather small and was from a single clinic of rheumatology, findings of this study cannot be generalized to all RA patients in our society. (3) The cross-sectional design of our study does not allow examining causal relationships between variables. Further studies with expanded sample sizes, objective sleep measures and prospective studies on RA patients’ quality of sleep should be conducted to support the development of effective interventions to improve their sleep quality.

## Conclusions

In summary, this is the first known evaluation of the contributors of poor sleep quality and the effects of sleep quality on RA patients’ HRQoL in China. The present study has found that the quality of sleep was very poor in RA patients and that poor sleep quality may significantly impair their HRQoL. The results of this research provided evidence of the importance of disease activity and depression in explaining sleep quality in patients with RA. The results emphasize the need for systemic psychiatric screening, holistic assessment and targeted intervention/management of RA patients to improve their sleep quality and finally improve their HRQoL.

## Additional file

Additional file 1: Table S1. Correlation coefficients between components of SF-36 and PSQI (Spearman rho). (DOC 58 kb)

## Abbreviations

ACR: American college of rheumatology
anti-CCP: anti-Cyclic Citrullinated Peptide
BMI: Body mass index
BP: Body pain
CRP: C-reactive protein
DAS28: Disease activity score in 28 joints
DMARD: Disease-modifying antirheumatic drugs
ELISA: Enzyme linked immunosorbent assay
ESR: Erythrocyte sedimentation rate
GH: General health perception
HADS: Hospital anxiety and depression scale
HAQ-DI: Health assessment questionnaire-disability index
HRQoL: Health-related quality of life
MCS: Mental components summary
MH: Mental health
NSAID: Nonsteroidal anti-Inflammatory Drugs
PCS: Physical components summary
PF: Physical function
PSQI: Pittsburgh sleep quality index
RA: Rheumatoid arthritis
RE: Role limitations due to emotional problems
RF: Rheumatoid factor
RP: Role limitations due to physical problems
SF: Social function
SF-36: The short form 36 health survey
VAS: Visual analog scale
VT: Energy/Vitality
