# Supplementary material for: Sleep quality in Chinese patients with rheumatoid arthritis: contributing factors and effects on health-related quality of life
Source: Health Qual Life Outcomes. 2016 Nov 16;14:151. doi: 10.1186/s12955-016-0550-3 (PMC5111274; doi:10.1186/s12955-016-0550-3)
Supplement: Additional file 1: Table S1. — Correlation coefficients between components of SF-36 and PSQI (Spearman rho). (DOC 58 kb) [file 12955_2016_550_MOESM1_ESM.doc]

Table S1 Correlation coefficients between components of SF-36 and PSQI (Spearman rho)

| **Variables** | **PF** | **RP** | **BP** | **GH** | **VT** | **SF** | **RE** | **MH** | **PCS** | **MCS** |
| --- | --- | --- | --- | --- | --- | --- | --- | --- | --- | --- |
| Female | -0.075 | -0.078 | 0.043 | 0.020 | 0.006 | -0.080 | -0.013 | -0.067 | -0.029 | -0.036 |
| BMI(kg/m^2^) | -0.047 | -0.171 | -0.164 | 0.001 | -0.117 | -0.153 | -0.105 | -0.052 | -0.130 | -0.130 |
| Age(years) | -0.111 | -0.116 | -0.129 | **0.191^*^** | 0.071 | **-0.203^*^** | -0.111 | 0.141 | -0.046 | -0.070 |
| Disease duration(years) | **-0.304^**^** | 0.062 | -0.125 | **-0.205^*^** | -0.127 | -0.093 | 0.056 | 0.013 | **-0.204^*^** | -0.042 |
| Monthly income(yuan) | **0.264^**^** | **0.307^**^** | **0.264^**^** | 0.138 | **0.188^*^** | **0.235^**^** | **0.186^*^** | **0.324^**^** | **0.319^**^** | **0.264^**^** |
| Marital status | -0.016 | 0.074 | -0.008 | **0.176^*^** | -0.046 | -0.037 | -0.006 | 0.103 | 0.046 | -0.0006 |
| Education | 0.057 | 0.114 | 0.157 | -0.021 | 0.023 | 0.055 | 0.077 | 0.099 | 0.119 | 0.080 |
| Occupation | -0.005 | 0.152 | 0.039 | 0.129 | 0.170 | 0.049 | 0.106 | **0.188^*^** | 0.094 | 0.122 |
| Smoke, yes | -0.074 | 0.023 | 0.066 | 0.047 | -0.004 | -0.116 | 0.081 | -0.062 | 0.041 | -0.020 |
| Alcohol, yes | -0.018 | -0.005 | -0.115 | **0.204^*^** | 0.061 | -0.101 | -0.054 | -0.021 | 0.025 | -0.065 |
| Family history | 0.098 | 0.069 | 0.055 | 0.031 | -0.040 | 0.111 | **0.187^*^** | 0.058 | 0.078 | 0.130 |
| Medications use | | | | | | | | | | |
| NSAIDs | -0.002 | -0.108 | -0.072 | 0.195^*^ | 0.157 | -0.042 | -0.089 | 0.059 | 0.015 | 0.011 |
| Synthetic DMARDs | -0.151 | **-0.221^*^** | **-0.192^*^** | -0.153 | -0.157 | **-0.183^*^** | **-0.173^*^** | -0.154 | **-0.199^*^** | **-0.189^*^** |
| Biologic DMARDs | 0.039 | 0.160 | -0.004 | 0.050 | 0.047 | 0.087 | 0.061 | 0.077 | 0.058 | 0.077 |
| Glucocorticoid | -0.135 | -0.113 | -0.139 | 0.078 | -0.086 | -0.167 | -0.052 | -0.014 | -0.103 | -0.101 |
| ESR(mm/h) | **-0.300^**^** | **-0.240^**^** | **-0.389^**^** | 0.031 | -0.124 | **-0.322^**^** | **-0.244^**^** | -0.061 | **-0.268^**^** | **-0.251^**^** |
| CRP(mg/dl) | **-0.342^**^** | **-0.294^**^** | **-0.437^**^** | -0.151 | -0.138 | **-0.361^**^** | **-0.284^**^** | **-0.183^*^** | **-0.379^**^** | **-0.306^**^** |
| RF positive | -0.166 | -0.135 | **-0.214^*^** | 0.050 | -0.092 | **-0.335^**^** | **-0.200^*^** | -0.170 | -0.138 | **-0.232^**^** |
| anti-CCP positive | -0.136 | -0.094 | -0.144 | 0.019 | 0.022 | **-0.220^*^** | -0.110 | -0.025 | -0.092 | -0.114 |
| DAS28 | **-0.349^**^** | **-0.377^**^** | **-0.541^**^** | **-0.336^**^** | **-0.216^**^** | **-0.389^**^** | **-0.328^**^** | **-0.256^**^** | **-0.484^**^** | **-0.375^**^** |
| HAQ-DI | **-0.729^**^** | **-0.496^**^** | **-0.644^**^** | **-0.399^**^** | **-0.443^**^** | **-0.546^**^** | **-0.340^**^** | **-0.413^**^** | **-0.738^**^** | **-0.519^**^** |
| Total pain(VAS) | **-0.386^**^** | **-0.368^**^** | **-0.701^**^** | **-0.286^**^** | **-0.362^**^** | **-0.390^**^** | **-0.267^**^** | **-0.308^**^** | **-0.550^**^** | **-0.391^**^** |
| Nocturnal pain (VAS) | **-0.393^**^** | **-0.382^**^** | **-0.674^**^** | **-0.202^*^** | **-0.305^**^** | **-0.462^**^** | **-0.373^**^** | **-0.345^**^** | **-0.526^**^** | **-0.462^**^** |
| HADS-A | **-0.362^**^** | **-0.345^**^** | **-0.412^**^** | **-0.413^**^** | **-0.470^**^** | **-0.412^**^** | **-0.457^**^** | **-0.557^**^** | **-0.490^**^** | **-0.570^**^** |
| HADS-D | **-0.338^**^** | **-0.387^**^** | **-0.373^**^** | **-0.204^*^** | **-0.473^**^** | **-0.455^**^** | **-0.338^**^** | **-0.437^**^** | **-0.421^**^** | **-0.487^**^** |
| PSQI | **-0.226^*^** | **-0.211^*^** | **-0.235^**^** | **-0.335^**^** | **-0.323^**^** | **-0.270^**^** | **-0.176^**^** | **-0.252^**^** | **-0.310^**^** | **-0.272^**^** |

**p* < 0.05; ***p* < 0.01.
